# Supplementary material for: Interactions between atomically dispersed copper and phosphorous species are key for the hydrochlorination of acetylene
Source: Commun Chem. 2022 Jan 10;5:2. doi: 10.1038/s42004-021-00619-7 (PMC9814576; doi:10.1038/s42004-021-00619-7)
Supplement: Supplementary file 1 — Supplementary Information [file 42004_2021_619_MOESM1_ESM.pdf]

Supplementary Information

Interactions between atomically dispersed copper and phosphorous species are key for the hydrochlorination of acetylene

Ting Wang[a], Zhao Jiang[a], Qi Tang[a], Bolin Wang[b], Saisai Wang[a], Mingde Yu[a], Renqin Chang[c], Yuxue Yue[a], Jia Zhao[a,*], Xiaonian Li[a,*]

[a] Industrial Catalysis Institute of Zhejiang University of Technology, Hangzhou, 310014, People's Republic of China

[b] School of Chemical Engineering, Northeast Electric Power University, Jilin, 132012, China

[c] Research Center of Analysis Measurement, Zhejiang University of Technology, Hangzhou, 310014, People's Republic of China

* Corresponding author. Tel: +86 571 88871656. E-mail: jiazhao@zjut.edu.cn (Jia Zhao)

* Corresponding author. Tel: +86 571 88320002. E-mail: xnli@zjut.edu.cn (Xiaonian Li)

Supplementary Table 1. C, Cu, P and O contents determined by XPS and EDS analysis over different samples.

Sample	XPS (wt.%)				EDS (wt.%)			
	C	P	Cu	O	C	P	Cu	O
Cu/PC200	51.00	13.20	8.17	27.63	55.51	12.06	10.41	22.02
Cu/PC400	46.00	13.81	9.17	31.02	54.04	12.38	8.14	25.44
Cu/PC600	56.72	11.58	8.39	23.32	57.06	11.81	9.88	21.25
Cu/PC800	76.51	5.25	9.33	8.91	74.95	6.32	10.37	8.36

Supplementary Table 2. ICP-AES analysis of copper in the catalysts and their activities in acetylene hydrochlorination.

Element	Cu/PC200	Cu/PC400	Cu/PC600	Cu/PC800
Cu	8.04%	8.87%	9.31%	8.73%

Supplementary Table 3. Textural properties of the phosphorus-doped carbon materials.

Sample	S_{BET} (m^2 g^{-1}) [a]	Volume (cm^{-3} g^{-1}) [b]	Diameter (nm) [c]
AC	1204	0.61	2.03
PC200	186	0.12	2.40
PC400	203	0.13	2.36
PC600	910	0.47	2.04
PC800	1005	0.58	2.19

[a] measured using the Brunauer-Emmett-Teller (BET) method; [b] calculated dependent on the adsorbed N_2 volume; [c] determined by Barrett-Joyner-Halenda (BJH) method.

Supplementary Table 4. Comparison of Cu-based catalyst in acetylene hydrochlorination.

Catalyst	Catalyst composition			Catalytic performance			Reference
	Active ingredient	Cu/wt.%	Carrier	Reaction conditions	Conversion/%	Selectivity/%	
Cu-g-C_3N_4/AC	Cu-Pyrrolic N	n.a.[a]	AC	180°C, 72 h^{-1}	79	>99.5	1
CuP/SAC	CuP	15	SAC	140°C, 180 h^{-1}	72	99.8	2
Cu-IL	Cu	0.7	n.a.[a]	180°C, 30 h^{-1}	65	>90	3
Cu/N-CNTs	Cu	5.84	N-CNTs	180°C, 180 h^{-1}	47	>98	4
Cu-Cs/AC	Cu-Cs	1	AC	200°C, 50 h^{-1}	92	>99	5
Cu/AC	Cu	5.02	AC	180°C, 30 h^{-1}	>90	n.a.[a]	6
Cu-HEDP/AC	Cu-HEDP	5	AC	180°C, 90 h^{-1}	>80	>99	7
Cu/SAC	Cu	15	SAC	180°C, 180 h^{-1}	98.7	>99.5	8
Cu-NMP/AC	Cu	15	AC	180°C, 160 h^{-1}	>89	n.a.[a]	9
Cu-HMPA/SAC	Cu-HMPA	15	SAC	180°C, 180 h^{-1}	87.25	>99	10
Cu-NMP/SAC	Cu	12	SAC	180°C, 36 h^{-1}	>99.9	>99.9	11
Cu/PC800	Cu-HEDP	10	AC	150°C, 180 h^{-1}	75	>99	This work

[a] n.a.: not available.

Supplementary Table 5. X-ray photoelectron spectroscopy (XPS) data of Cu 2p spectra of fresh P-doped Cu-based catalysts from this work.

Sample	Cu^+/Cu^0 [a]			Cu^{2+} [a]		
	Position/ eV	FWHM[b]/ eV	Area/ %	Position/ eV	FWHM[b]/ eV	Area/ %
Cu/PC200	932.4	2.25	71.4	934.6	2.91	28.6
Cu/PC400	932.4	2.20	67.7	934.5	2.91	32.3
Cu/PC600	932.4	2.15	64.9	934.5	3.22	35.1
Cu/PC800	932.3	2.15	27.4	934.6	2.41	72.6

[a] based on reference values; [b] FWHM = full width at half maximum.

Supplementary Table 6. X-ray photoelectron spectroscopy (XPS) data of Cu XAES spectra of fresh P-doped Cu-based catalysts from this work.

Sample	Cu^{0} [a]			Cu^{+} [a]		
	Position/ eV	FWHM[b]/ eV	Area/ %	Position/ eV	FWHM[b]/ eV	Area/ %
Cu/PC200	918.6	3.78	16.6	916.6	4.13	54.8
Cu/PC400	918.6	3.61	11.5	916.6	4.21	56.2
Cu/PC600	918.7	3.63	7.6	916.6	4.22	57.3
Cu/PC800	918.6	3.61	3.6	916.6	4.28	23.8

[a] based on reference values; [b] FWHM = full width at half maximum.

Supplementary Table 7. X-ray photoelectron spectroscopy (XPS) data of P 2p spectra of fresh P-doped Cu-based catalysts from this work.

Sample	P-C [a]			P-O [a]			P=O [a]		
	Position/ eV	FWHM[b]/ eV	Area/ %	Position/ eV	FWHM[b]/ eV	Area/ %	Position/ eV	FWHM[b]/ eV	Area/ %
Cu/PC200	133.0	1.32	15.6	134.2	1.77	71.2	135.2	1.94	13.2
Cu/PC400	133.0	1.50	27.4	134.3	1.96	59.9	135.2	1.96	12.7
Cu/PC600	133.1	1.28	33.7	134.1	1.63	54.5	135.1	1.96	11.8
Cu/PC800	133.0	1.52	41.6	134.3	1.60	48.3	135.1	1.95	10.1

[a] based on reference values; [b] FWHM = full width at half maximum.

Supplementary Table 8. X-ray photoelectron spectroscopy (XPS) data of O 1s spectra of fresh P-doped Cu-based catalysts from this work.

Sample	P=O [a]			P-O-C [a]		
	Position/ eV	FWHM[b]/ eV	Area/ %	Position/ eV	FWHM[b]/ eV	Area/ %
Cu/PC200	531.5	1.62	29.6	532.6	2.29	70.4
Cu/PC400	531.3	1.80	28.2	532.4	2.60	71.8
Cu/PC600	531.4	1.73	26.2	532.5	2.40	73.8
Cu/PC800	531.3	1.66	25.3	532.5	2.48	74.7

[a] based on reference values; [b] FWHM = full width at half maximu

Supplementary Table 9. Fitting parameters from the EXAFS spectra of the selected catalysts.

Sample	Scattering Path	CN[a]	R(Å)[b]	$\sigma^2\times10^{-3}$(Å^2)[c]	R factor (%)
Cu foil	Cu-Cu	12	2.55±0.01	8.9±0.5	0.32
$CuCl_2$	Cu-Cl	3.7±0.3	2.16±0.01	11.0±1.1	0.82
Cu/PC800	Cu-Cl	3.8±0.3	2.17±0.01	11.1±1.1	0.84

[a] Coordination number. [b] Coordination shell distance. [c] Debye-Waller factor.

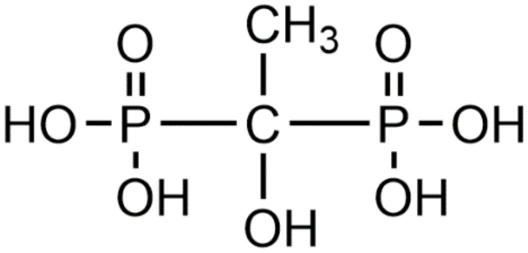

Supplementary Figure 1. The structure of HEDP.

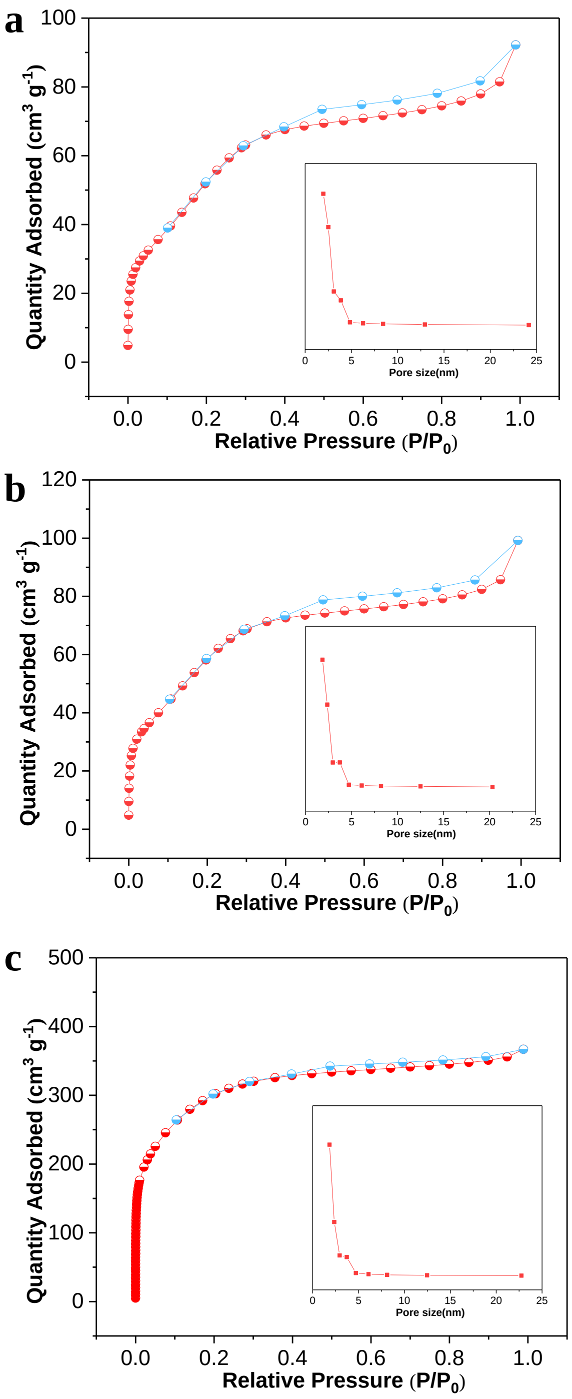

Supplementary Figure 2. N_2 gas adsorption / desorption isotherms of **a** PC200, **b** PC400 and **c** PC600.

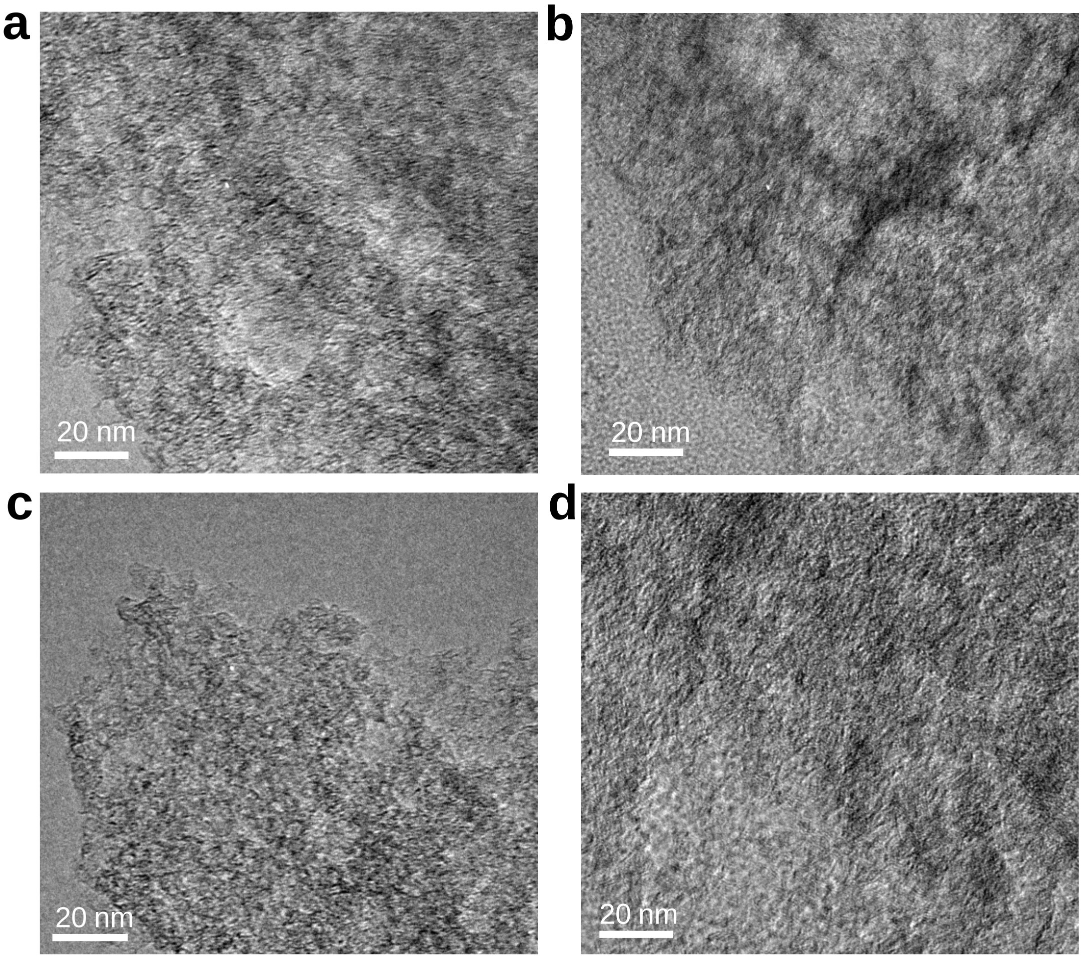

Supplementary Figure 3. HRTEM image of **a** Cu/PC200, **b** Cu/PC400, **c** Cu/PC600 and **d** Cu/PC800 catalysts.

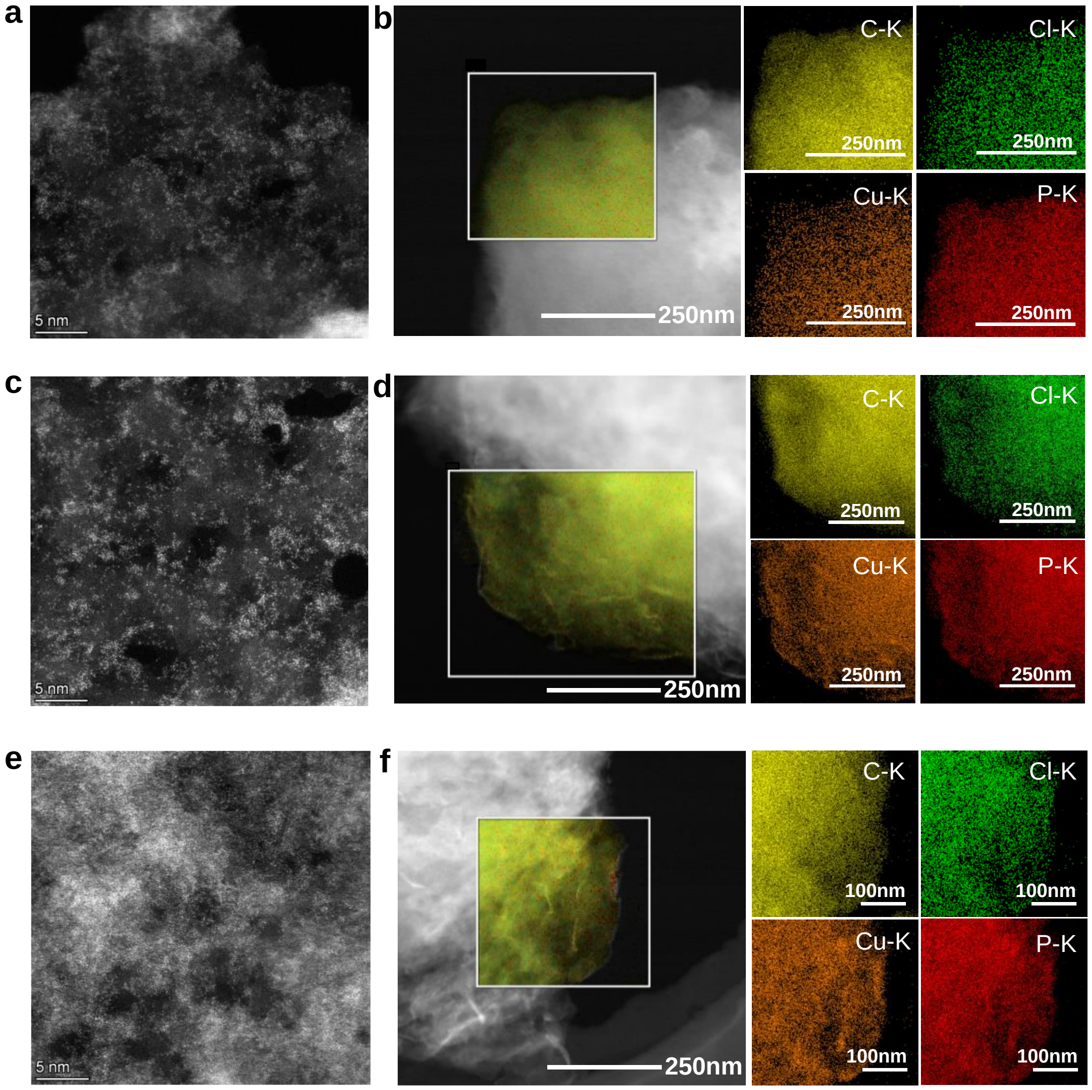

Supplementary Figure 4. a Representative HAADF-STEM image and **b** EDS elemental mapping of fresh Cu/PC200, **c** Representative HAADF-STEM image and **d** EDS elemental mapping of fresh Cu/PC400, **e** Representative HAADF-STEM image and **f** EDS elemental mapping of fresh Cu/PC600.

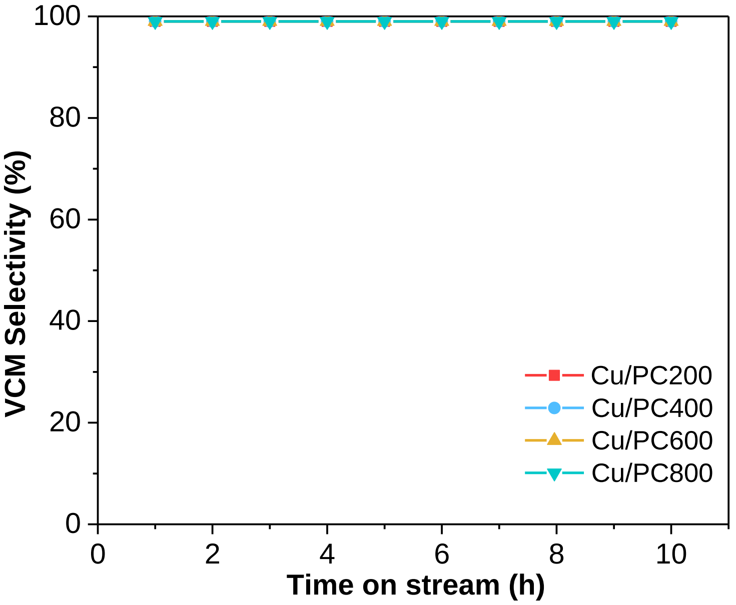

Supplementary Figure 5. Selectivity to VCM over P-doped Cu-based catalysts.

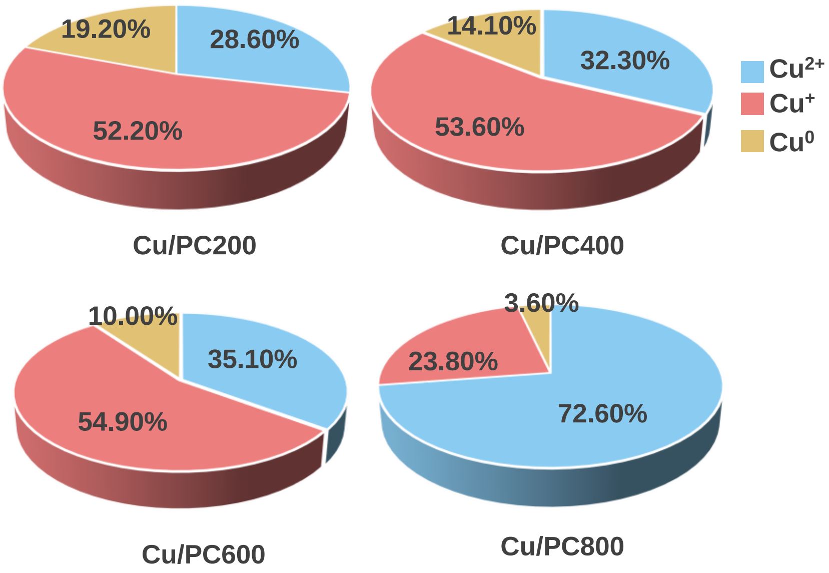

Supplementary Figure 6. Corresponding-Cu contents of fresh P-doped Cu-based catalysts.

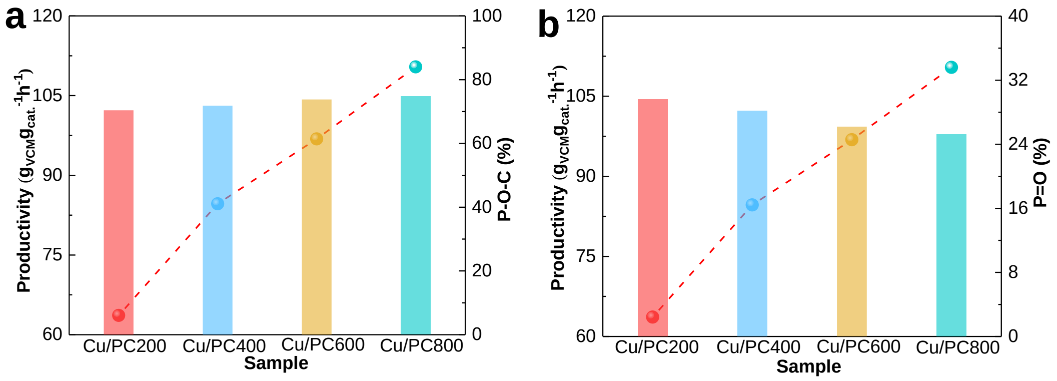

Supplementary Figure 7. Correlation diagram of oxygen species content and productivity.

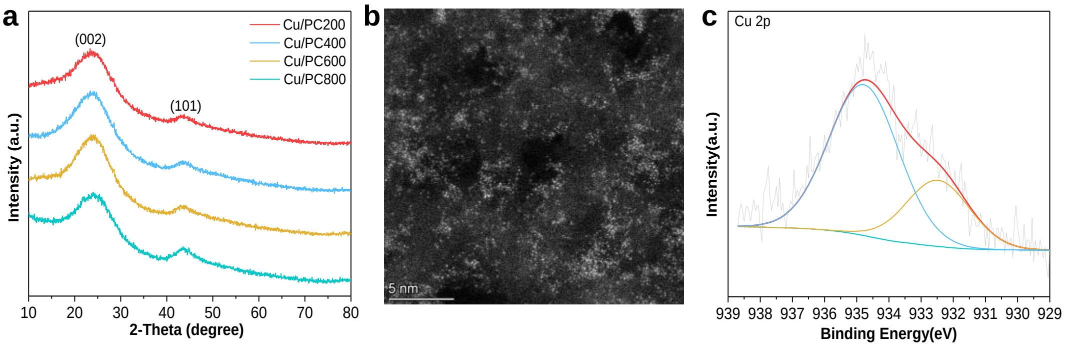

Supplementary Figure 8. a XRD pattern of used P-doped Cu-based catalysts, **b** Representative HAADF-STEM image and **c** Cu 2p XPS spectra of the used Cu/PC800.

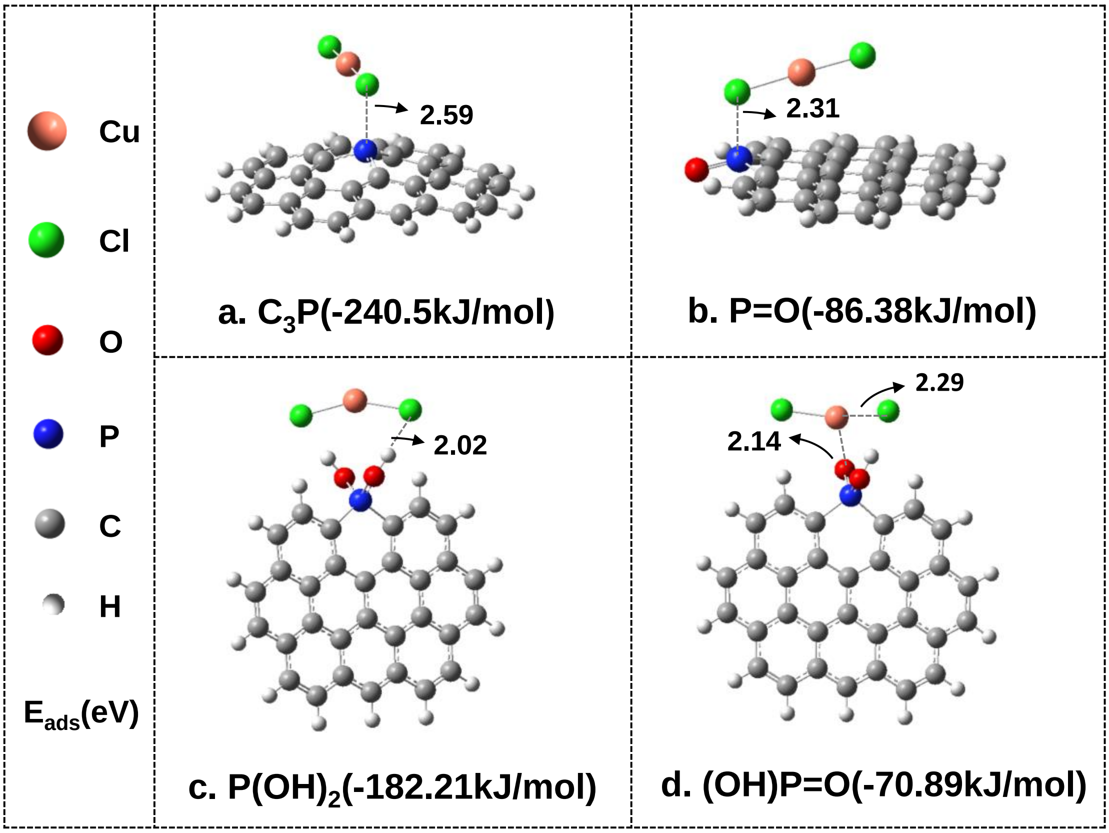

Supplementary Figure 9. Optimized geometries of Cu^{2+} active sites adsorption on **a** C_3P, **b** P=O, **c** $P(OH)_2$, **d** (OH)P=O, respectively. Numbers in black are distances (Å) between atoms.

Supplementary References

1. Zhao, W., Zhu, M. & Dai, B. The preparation of Cu-g-C_3N_4/AC catalyst for acetylene hydrochlorination. *J. Catal*. **6**, 193-203 (2016).

2. Li, H., Wang, F., Cai, W., Zhang, J. & Zhang, X. Hydrochlorination of acetylene using supported phosphorus-doped Cu-based catalysts. *Catal. Sci. Technol.* **5**, 5174-5184 (2015).

3. Qin, G. et al. Gas-liquid acetylene hydrochlorination under nonmercuric catalysis using ionic liquids as reaction media. *Green Chem*. **13**, 1495-1498 (2011).

4. Zhou, K. et al. Reactivity enhancement of N-CNTs in green catalysis of C_2H_2 hydrochlorination by a Cu catalyst. *RSC Adv*. **4**, 7766-7769 (2014).

5. Zhai, Y. et al. Carbon-supported perovskite-like $CsCuCl_3$ nanoparticles: a highly active and cost-effective heterogeneous catalyst for the hydrochlorination of acetylene to vinyl chloride. *Catal. Sci. Technol.* **8**, 2901-2908 (2018).

6. Xu, H., Si, J. & Luo, G. The kinetics model and fixed bed reactor simulation of Cu catalyst for acetylene hydrochlorination. *Int. J. Chem. React. Eng*. **15**, 20160165 (2017).

7. Wang, X., Zhu, M. & Dai, B. Effect of phosphorus ligand on Cu-based catalysts for acetylene hydrochlorination. *ACS Sustain. Chem. Eng*. **7**, 6170-6177 (2019).

8. Wang, Y., Nian, Y., Zhang, J., Li, W. & Han, Y. MOMTPPC improved Cu-based heterogeneous catalyst with high efficiency for acetylene hydrochlorination. *Mole. Catal*. **479**, 110612 (2019).

9. Zhao, C., Zhang, X., He, Z., Guan, Q. & Li, W. Demystifying the mechanism of NMP ligands in promoting Cu-catalyzed acetylene hydrochlorination: insights from a density functional theory study. *Inorg. Chem. Front*. **7**, 3204-3216 (2020).

10. Hu, Y. et al. High performance of supported Cu-based catalysts modulated via phosphamide

coordination in acetylene hydrochlorination. *Appl. Catal. A Gen.* **591**, 117408 (2020).

11. Han, Y. et al. Pyrrolidone ligand improved Cu-based catalysts with high performance for acetylene hydrochlorination. *Appl. Organomet. Chem.* **35**, e6066 (2020).
